# Benzoindolizidine Alkaloids Tylophorine and Lycorine and Their Analogues with Antiviral, Anti-Inflammatory, and Anticancer Properties: Promises and Challenges

**DOI:** 10.3390/biomedicines11102619

**Published:** 2023-09-24

**Authors:** Antonella Di Sotto, Mehdi Valipour, Aala Azari, Silvia Di Giacomo, Hamid Irannejad

**Affiliations:** 1 Department of Physiology and Pharmacology “V. Erspamer”, Sapienza University of Rome, P.le Aldo Moro 5, 00185 Rome, Italy; silvia.digiacomo@iss.it; 2 Razi Drug Research Center, Iran University of Medical Sciences, Tehran 14496-14535, Iran; 3 Environment and Health, Department of Public Health and Primary Care, KU Leuven, Herestraat 49, 3000 Leuven, Belgium; aala.azari@kuleuven.be; 4 Department of Food Safety, Nutrition and Veterinary Public Health, National Institute of Health, 00161 Rome, Italy; 5 Department of Medicinal Chemistry, Faculty of Pharmacy, Mazandaran University of Medical Sciences, Sari 48471-93698, Iran; irannejadhamid@gmail.com

**Keywords:** benzoindolizidine alkaloids, lycorine, tylophorine, anti-inflammatory, antiviral, SARS-CoV-2, NF-κB

## Abstract

Ongoing viral research, essential for public health due to evolving viruses, gains significance owing to emerging viral infections such as the SARS-CoV-2 pandemic. Marine and plant alkaloids show promise as novel potential pharmacological strategies. In this narrative review, we elucidated the potential of tylophorine and lycorine, two naturally occurring plant-derived alkaloids with a shared benzoindolizidine scaffold, as antiviral agents to be potentially harnessed against respiratory viral infections. Possible structure-activity relationships have also been highlighted. The substances and their derivatives were found to be endowed with powerful and broad-spectrum antiviral properties; moreover, they were able to counteract inflammation, which often underpins the complications of viral diseases. At last, their anticancer properties hold promise not only for advancing cancer research but also for mitigating the oncogenic effects of viruses. This evidence suggests that tylophorine and lycorine could effectively counteract the pathogenesis of respiratory viral disease and its harmful effects. Although common issues about the pharmacologic development of natural substances remain to be addressed, the collected evidence highlights a possible interest in tylophorine and lycorine as antiviral and/or adjuvant strategies and encourages future more in-depth pre-clinical and clinical investigations to overcome their drawbacks and harness their power for therapeutic purposes.

## 1. Introduction

Alkaloids are a huge group of plant metabolites characterized by diverse and often complex structures, typically containing a basic nitrogen atom derived from an amino acid [1]. They are widely distributed in nature, being especially abundant in plant families, including, but not limited to, Amaryllidaceae, Papaveraceae, and Solanaceae [2]. In plants, alkaloids serve a dual role as allelopathic agents, protecting plants from predators, and as growth regulators [1].

Numerous studies have unveiled the healing properties of some alkaloids in humans, leading to the discovery of noteworthy compounds harnessed for medicinal purposes; among them, vincristine and vinblastine (anticancer agents), reserpine (an antihypertensive agent), physostigmine (a cholinesterase inhibitor), and ajmaline (an anti-arrhythmic agent) are notable examples [3]. Other compounds, such as taxol (a diterpene pseudoalkaloid) from Pacific Yew (*Taxus brevifolia* Nutt.) bark, morphine (a phenanthrene alkaloid) from opium poppy (*Papaver somniferum* L.), and camptothecin (a quinolone alkaloid) from *Camptotheca acuminata* Decne. bark and stem, served as inspiring structures and scaffolds for drug discovery [1]. Over the years, great attention has been devoted to the role of alkaloids in fighting viral diseases, which pose a permanent challenge not only for humans but also for plants and animals [4].

Viruses exhibit complex mechanisms for infiltrating host cells, replication, and evading immune responses; additionally, their rapid mutation rates and capacity to generate new variants make it challenging to effectively control their pathogenic potential [4]. Viral pathogenesis is influenced by several factors, including cellular tropism, which is a critical factor in the onset and manifestation of viral infections, as well as the host immune response, which determines the progression and severity of viral diseases [4]. Usually, the immune response is able to lead to infection resolution and recovery; however, some viruses have evolved mechanisms to evade or subvert these host defenses, thus causing prolonged viral persistence, chronic infections, and complications in other sites, like liver damage and cancer [5]. Certain viruses, such as rabbit papilloma virus (HPV), Epstein–Barr virus (EBV), hepatitis B and C viruses (HBV and HCV), and human T-cell leukemia virus type 1 (HTLV-1), play a role in the development of both benign and malignant tumors [6]. Some studies also highlighted an association between respiratory infections and lung cancer development; particularly, cumulative exposure to influenza viruses seems to increase the risk of lung cancer, both in animal models and population studies [7,8,9,10]. Interestingly, a common involvement of the angiotensin converting enzyme 2 (ACE2) in both lung cancer and coronavirus infections, such as severe acute respiratory syndrome coronavirus 2 (SARS-CoV-2) (COVID-19), has been reported [11,12,13,14].

This evidence strengthens the crucial need for tailored approaches to the prevention, diagnosis, and treatment of viral infections and suggests the importance of interdisciplinary research and collaboration to address the complex challenges posed by viral pathogens with respect to not only the immediate outcomes but also the long-term effects, such as the risk of initiating carcinogenesis in different organs.

In this respect, natural substances offer intriguing and promising opportunities for new structures and mechanisms to be harnessed in the battle against viral infections and their complications, although the clinical evidence in support is still limited [15,16,17,18]. Among natural substances, several alkaloids, such as berberine, matrine, homoharringtonine, and aloperine, showed antiviral properties in vitro [19,20,21,22,23]. Moreover, other ones, which include caffeine, ergotamine, amaranthin, indirubin, sanguinarine, solanidine, and thebaine, have been highlighted to possess anti-SARS-CoV-2 activities in silico [24]. In our previous study, we highlighted the promising role of isoquinoline-containing alkaloids, especially emetine, cephaeline, and papaverine, as anti-SARS-CoV-2 agents, arising from their dual antiviral and anti-inflammatory properties [25]. Moreover, these alkaloids were able to inhibit the p38 MAPK signaling pathway, which holds promise as a host-based anti-COVID-19 target.

**Figure 1:**
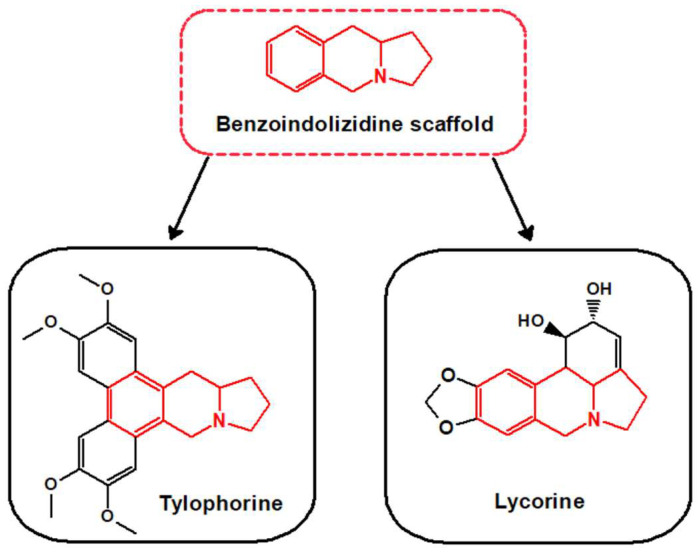
Benzoindolizidine scaffold and the representative compounds tylophorine and lycorine (drawn by ChemSketch, ACD Labs Version 12.0).

In line with this evidence, in the present review, we focused our attention on benzoindolizidine alkaloids, a subgroup of the indolizidine alkaloids [26], containing an indolizidine moiety (a 5-membered ring fused with a 6-membered one with a common nitrogen atom) linked to a benzene ring (Figure 1). This peculiar scaffold has attracted great attention since it has been associated with several biological activities, including anticancer, anti-inflammatory, immunomodulatory, and antiviral ones [27]. Tylophorine and lycorine (Figure 1) have recently emerged as representative benzoindolizidine alkaloids, endowed with promising bioactivities that could potentially be harnessed in the battle against viral infections and their complications.

Considering the above, the aim of this present narrative review is to provide an overview of the existing preclinical and clinical evidence regarding the bioactivity profile of both tylophorine and lycorine, while also focusing on their underlying mechanisms of action. Furthermore, insights into their natural occurrence, chemical features, pharmacokinetic properties, and safety profile are given. Lastly, we discuss current challenges and future perspectives related to the potential utilization of these compounds as antiviral strategies.

The literature search was performed using PubMed and SCOPUS electronic databases without time limitations, and establishing English as the preferred language. Moreover, for more specific requirements, Google Scholar and ClinicalTrials.gov were considered. The following searching keywords and their combinations through the Boolean logical operators have been used: “tylophorine”, “lycorine”, “benzoindolizidine alkaloids”, “natural occurrence”, “antiviral”, “anticancer”, “COVID-19”, “chemical features”, “preclinical studies”, “in vitro”, “in vivo”, “clinical trials”, “anti-inflammatory”, “protection”, “apoptosis”, “apoptotic signaling”, “PI3K”, “Akt”, “mTOR”, “Nrf2”, “NF-κB”, “inflammation”, “pharmacokinetic”, and “bioavailability”. The up-to-date studies focused on the antiviral, anti-inflammatory, and anticancer effects of tylophorine and lycorine and their mechanisms of action have been included in the review. Conversely, those regarding the bioactivities of herbal extracts or other natural sources containing them, but not the pure compounds, were excluded.

## 2. Tylophorine and Its Derivatives

### 2.1. Chemistry and Occurrence in Nature

**Figure 2:**
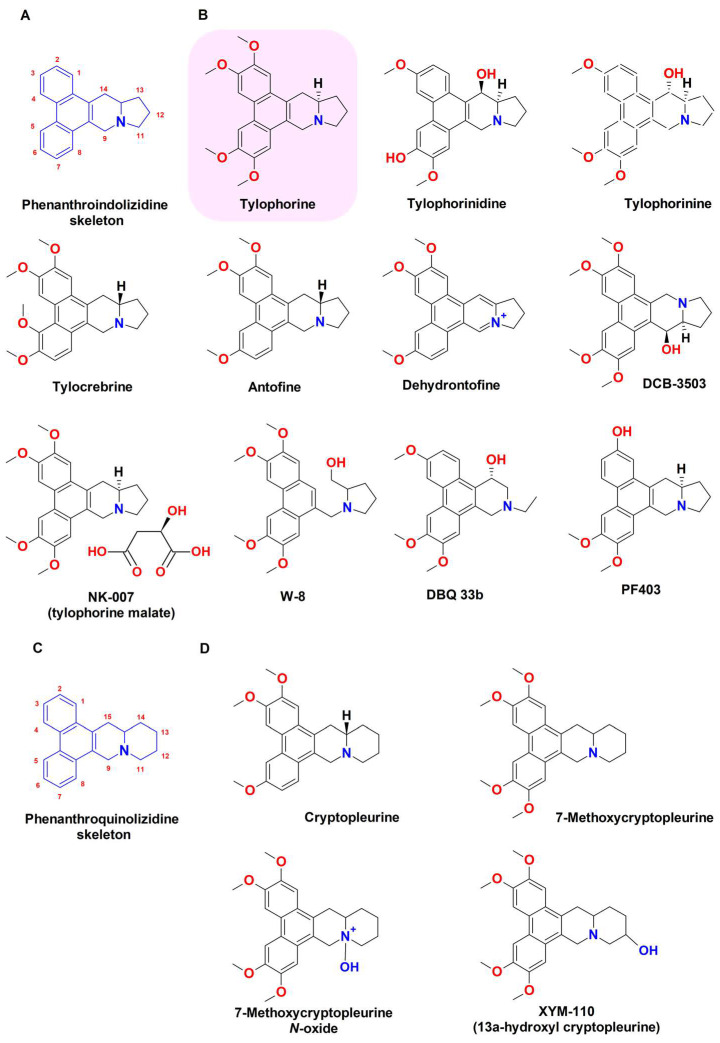
Chemical structures of tylophorine-based alkaloids. (**A**) Phenanthroindolizidine skeleton, containing a highly oxygenated phenanthrene group fused with an indolizidine group. (**B**) Tylophorine and its phenanthroindolizidine derivatives. (**C**) Phenanthroquinolizidine skeleton, containing a highly oxygenated phenanthrene group fused with an indolizidine one. (**D**) Phenanthroquinolizidine alkaloids (drawn by ChemSketch, ACD Labs Version 12.0).

Tylophorine (C_24_H_27_NO_4_; molecular weight 393.19 g/moL) is a naturally occurring alkaloid (Figure 1) belonging to the phenanthroindolizidine class, characterized by a pentacyclic structure with a highly oxygenated phenanthrene skeleton fused with an indolizidine group (Figure 2A); a similar structure is shared by phenanthroquinolizidine alkaloids, containing a quinolizidine instead of the indolizidine moiety (Figure 2C) [28]. Different phenanthroindolizidine and phenanthroquinolizidine alkaloids occur in nature, mainly in plants from the Asclepiadaceae (now a subfamily of the Apocynaceae family) and Moraceae families [29,30]. Among them, tylophorine and its structural analogues tylophorinine and tylophorinidine (Figure 2B), also known as tylophora alkaloids (Figure 2), have been identified firstly in 1935 by Ratnagiriswaran and Venkatachalam in leaves and roots (about 0.2 to 0.46% *w*/*w*) of *Tylophora indica* (Burm. F.) Merr. (syn. *Tylophora asthmatica* (L. f.) Wight & Arn., *Asclepias asthmatica* L. f.; fam. Asclepiadaceae), a perennial plant widely distributed in Asia, Africa, Australia, and Oceanic islands and commonly known as “Indian ipecacuanha” or “emetic swallow-wort” [31,32].

Further studies highlighted the presence of these compounds and other tylophorine-based alkaloids (e.g., tylocrebrine) in *Tylophora* sp. (e.g., *Tylophora crebriflora* S.T. Blake, *Tylophora ovata* (Lindl.) Hook. Ex Steud., *Tylophora mollissima* Wt., *Tylophora tanakae* Maxim. Ex Franch. & Sav.) (Table 1). Moreover, tylophorine-based alkaloids, such as antofine and cryptopleurine (Figure 2D), have been isolated from other species, which include *Ficus septica* Burm. F. (Moraceae), *Cryptocarya laevigata* Blume (Lauraceae), and *Cynanchum komarovii* Al. Iljinski (Asclepiadaceae) (Table 1). Their levels in plants can differ due to several factors, such as species, harvesting time, and cultivation conditions. For instance, the roots and leaves of *T. indica* contain about 0.2–0.46% *w*/*w* tylophorine, tylophorinine, and tylophrinidine [33], with tylophorine ranging from 0.01 to 0.16% *w*/*w* in leaves and about 0.08% *w*/*w* in roots [34,35,36].

Since their first isolation, natural phenanthroindolizidine and phenanthroquinolizidine alkaloids have attracted great scientific interest owing to their diverse bioactivities [28]. Indeed, some studies have highlighted the anti-allergic activity of tylophorine, likely ascribable to its immunosuppressive effects on mast cell degranulation and T cell-mediated responses and to its direct bronchodilator and membrane stabilizing properties [28]. Moreover, tylophorine-based compounds have been shown to possess antimicrobial, insecticidal, hypolipidemic, antiviral, anti-inflammatory, and anticancer properties [28,44]. In order to increase their potency and selectivity and to lower toxicity, a number of synthetic and shemisynthetic derivatives, such as DCB-3503 (a hydroxylated analogue of tylophorine) and 7-methoxycryptopleurine, have been developed; however, none have been clinically developed [29,45,46]. In the following paragraphs, the antiviral, anti-inflammatory, and anticancer properties of tylophorine and tylophorine-based alkaloids will be deeply discussed, based on the large number of studies present in the literature.

### 2.2. Antiviral Activity

In the last decades, special attention has been focused on the antiviral properties of phenanthroindolizidine and phenanthroquinolizidine alkaloids against different strains, mainly belonging to the Coronavirus, Tobamovirus, Lentivirus, and Hepacivirus genera [28]. Tylophorine and its natural or synthetic derivatives have been mainly studied; details about natural sources and chemical structures are reported in Table 1 and Figure 2.

Yang et al. [46] reported for the first time the anticoronaviral properties of tylophorine and its analogues (both natural and synthetic) against the porcine transmissible gastroenteritis coronavirus (TGEV) and human severe acute respiratory syndrome coronavirus (SARS-CoV), belonging to Group I and II of coronaviruses, respectively. Among the tested alkaloids, tylophorine, tylophorinine, and 7-methoxycryptopleurine produced anti-TGEV effects in swine testicular (ST) epithelial cells, although higher anti-TGEV potencies were found for tylophorine-based derivatives [46]. This evidence was corroborated through a comparison of the EC50 (50% maximal effective concentration) values and selective index (SI), as determined in the cytopathic assay (Table 2). Similarly, tylophorine-derived dibenzoquinolines exhibited enhanced antiviral effects against TGE with respect to tylophorine while demonstrating reduced neurotoxicity. Moreover, combining the tylophorine-based compounds with a JAK2 (Janus Kinase 2)/NF-κB (nuclear factor kappa B) inhibitor has been highlighted as a possible more effective strategy to treat coronavirus infections [47].

Tylophorine, tylophorinine, and 7-methoxycryptopleurine oxide strongly inhibited SARS-CoV (Urbani strain) infection in Vero 76 cells, with EC50 values ranging from <5 to 18 nM and SI from 78 to >100. An antiviral activity of tylophorine against MHV (mouse hepatitis virus) β-coronavirus has also been reported, although details are not available. Recently, the authors demonstrated the anticoronaviral properties of tylophorine and its dibenzoquinoline derivatives (containing diverse N-substituents or a hydroxyl group at the C14 position) against a variety of viral strains, including the feline inflammatory peritonitis virus (FIPV) and the human coronaviruses HCoV-OC43, HCoV-229E, and SARS-CoV-2. All the tested compounds were able to potently inhibit both HCoV-OC43 and SARS-CoV-2 strains (Table 2), thus suggesting a further interest in the battle against the COVID-19 pandemic [48].

The anticoronaviral properties of tylophorine and Its derivatives are ascribed to their ability to directly target the viral replication-transcription machinery by interacting with genomic/subgenomic RNA and nucleocapsid proteins, colocalizing with coronaviral RNA and RNA-dependent RNA polymerase in the viral replication transcription complexes (RTCs), and blocking the syntheses of coronaviral antigens in genomic/subgenomic RNAs [48]. Considering the crucial role of N proteins and genomic/subgenomic RNA complexes in coronavirus replication, the multitargeted activity of tylophorine-based compounds could represent an interesting anticoronaviral strategy to be further investigated and evaluated clinically.

Regarding the structure-activity relationship (SAR), Yang et al. [46] highlighted that the non-planar structure of the indolizidine moiety was crucial for the anti-TGEV effects. Usually, the presence of hydroxyl substituents at carbon 14 (C14) of the indolizidine moiety (Figure 2A) and at C3 of the phenanthrene group potentiates the anti-TGEV effects of tylophorine, while adding a methoxyl substituent at C2, a carbonyl at C9, or an acetoxyl one at C14 decreases the antiviral activity. Moreover, *N*-oxide derivatives seem to be less effective than the lead compound, except for the quinolizidine derivatives (Figure 2C,D): indeed, 7-methoxy cryptopleurine *N*-oxide (Figure 2C) was found to be more potent as an anti-SARS-CoV than its indolizine counterpart [46].

Tylophorine-based alkaloids, which include (*R*)-antofine and (*R*)-tylophorine (Figure 2B), and phenanthroquinolizidine alkaloids, such as cryptopleurine (Figure 2D), also produced strong antiviral effects in vitro against tobacco mosaic virus (TMV; Tobamovirus genus), which are more potent than ningnanmycin and ribavirin and are usually used as antiviral drugs to treat TMV infection in plants [45,49]. The activity was maintained by C9-substituted phenanthrene-based compounds and salt derivatives, which were also more stable and water-soluble than tylophorine, thus enabling it to overcome its major drawbacks [43,46].

The anti-TMV activity of tylophorine-based compounds has been ascribed to their ability to interfere with the initiation of virus assembly and to disrupt the interaction between TMV RNA and coat protein [43,48]. SAR studies highlighted the non-planar indolizidine moiety and the presence of hydroxyl and alkoxyl groups in the phenanthrene unit as structural requirements for the anti-TMV activity; moreover, α-hydroxymethyl substituents greatly enhance the antiviral power; and at last, nitrogen salinization increases the compound stability and water solubility [45,49].

Dehydroantofine (Figure 2B) was also found to inhibit in vitro the replication of human immunodeficiency virus (HIV; Lentivirus genus) in human T cell line H9 cells (EC50 1.88 μg/mL), despite a null effect of (*R*)-antofine [50]. Furthermore, the tylophorine analogues DCB-3503 and rac-cryptopleurine were reported to strongly inhibit hepatitis C virus (HCV; Hepacivirus genus) replication in Huh-luc/neo-ET cells [51].

This antiviral activity seems to be due to an allosteric regulation of the ATPase activity of heat shock cognate protein 70 (Hsc70), involved actively in HCV replication; consequently, translation of HCV RNA is perturbed, thus leading to the inhibition of HCV replication [51]. The anti-HCV activity of rac-cryptopleurine was enhanced by promoting the chaperone activity of Hsc70; the 13-hydroxyl cryptopleurine derivative XYM-110 (Figure 2D) maintained the anti-HCV activity of the lead compound and promoted the ATP/ADP turnover of Hsc70, while hydroxylated analogues at other positions exhibited a lower antiviral power [52].

**Table 2:** In vitro studies on the anticoronaviral properties of tylophorine and its natural and synthetic derivatives.

Viral Strain (Viral Genus)	Infected Cells	EC50	Selective Index (SI)	Ref.
Tylophorine				
TGEV (porcine coronavirus)	ST	58 nM	>1000	[46]
	ST	80 nM	-	[53]
MHV (mouse hepatitis virus; β-coronavirus)	-	-	-	[53]
FIPV (Feline coronavirus)	Fcwf-4	62 nM	>100	[48]
SARS-CoV (human coronavirus)	Vero 76	18 nM	88	[46]
HCoV-OC43, HCoV-229E, SARS-CoV-2 (human coronavirus)	HCT-8	68 to 78 nM	46 to >100	[48]
Tylophorine-based derivatives				
TGEV (porcine coronavirus)	ST	8 to 18 nM	>7 to >1000	[46]
	ST	0.6 to >50 μMb	-	[53]
	ST	0.04 to >2.9 μM	-	[53]
FIPV (Feline coronavirus)	Fcwf-4	8 to >1000 nM	12.3 to >100	[48]
SARS-CoV (human coronavirus)	Vero 76	<5 to 340 nM	10 to >100	[46]
HCoV-OC43, HCoV-229E, SARS-CoV-2 (human coronavirus)	HCT-8	2.5 to 78 nM	>5.3 to >100	[48]
Tylophorinine				
TGEV (porcine coronavirus)	ST	82 nM	>1000	[46]
SARS-CoV (human coronavirus)	Vero 76	<5 to 18 nM	78 to >100	[46]
7-Methoxy cryptopleurine				
TGEV (porcine coronavirus)	ST	20 nM	>1000	[46]
	ST	30 nM	-	[53]
SARS-CoV (human coronavirus)	Vero 76	39 nM	19	[46]

ST, swine testicular epithelial cells; Vero 76, African green monkey kidney cells; Fcwf-4, felis catus whole fetus-4 cells; HCT-8, human colon adenocarcinoma cells; TGEV, porcine transmissible gastroenteritis coronavirus; FIPV, Feline infectious peritonitis virus.

### 2.3. Anti-Inflammatory Activity

Tylophorine-based alkaloids, either naturally occurring or chemically synthesized, exhibited anti-inflammatory properties in different preclinical models [28,54]. Particularly, *R*-tylophorine and *R*-antofine showed to suppress the lipopolysaccharide (LPS)- and interferon (IFN)-γ-induced release of proinflammatory factors, especially nitric oxide (NO), in RAW 264.7 macrophages, with a higher potency of tylophorine. The presence of methoxy groups at the C2, C3, C6, and C7 positions of the phenanthroindolizidine skeleton (Figure 2A) of tylophorine was found to be mandatory for its bioactivity, while the substitution of the methoxy group at C3 and C6 lowered the potency [28]; these chemical features were also confirmed for the antofine derivatives. Introducing a carbonyl group at C9 decreased the anti-inflammatory potency, likely due to an increase in the rigidity and planarity of the indolizidine moiety [28]. A marked anti-inflammatory potency has also been displayed by 7-methoxycryptopleurine (Figure 2C), suggesting that the phenanthroquinolizidine skeleton (Figure 2B) was more potent as an NO suppressor than its phenanthroindolizidine counterpart [28]. According to the chemical features previously described for the antiviral activity, the non-planar structure of the indolizidine moiety has a crucial role for the anti-inflammatory activity: indeed, increasing rigidity and planarity greatly reduced the bioactivity [28]. Salinization of DCB-3503 and tylophorine enhanced the efficacy of the compounds, likely by improving their water solubility [55].

Along with the NO suppression, *R*-antofine has been shown to inhibit the production of iNOS (inducible nitric oxide synthase) and the secretion of tumor necrosis factor α (TNF-α) and interleukin (IL) 1β in LPS-activated RAW 264.7 cells; these effects were associated with a lowered expression of different genes, which include the vasopressor *EDN1* (endothelin-1), and some related to inflammation (such as arginase 1 or *ARG-1*, *IL-1F9*, *IL-10*, and *IL-33*) and to extracellular matrix (such as tenascin C or *TNC* and hyaluronidase 1 or *HYAL1*). Furthermore, the compound modulated the activation of AMPK and caspase-1, which regulate inflammasome-mediated IL-1β maturation [56].

The tylophorine analogue DCB-3503 (Figure 2B) and its C9-substituted derivative also regulated the differentiation of T cells (Tregs); this effect seems to arise from a Forkhead Box Protein P3 (Foxp3) transcription factor stimulation, associated with an enhanced demethylation of its promoter induced by the inhibition of ERK (extracellular signal-regulated kinase) and DNMT1 (DNA methyltransferase 1) expression [54]. Moreover, interference with the AKT (AK mouse plus Transforming or Thymoma)/mTOR (mechanistic target of rapamycin kinase) pathway has been reported [55]. A similar behavior has been highlighted for the more stable and low-toxic tylophorine derivative W-8 in primary CD4+ T cells [57].

Furthermore, the compounds inhibited the production of TNF-α in RAW 264.7 macrophages and primary murine splenocytes from BALB/c mice [55]. An inhibition of the NF-κB signaling pathway, associated with a down-regulation of nuclear phosphorylated p65, seems to be also involved in the anti-inflammatory activity of DCB-3503 [58]. Accordingly, (±)-tylophorine malate NK-007 (Figure 2B) decreased phosphorylation of NF-κB, TNF-α, and IL-12 in RAW 264.7 and murine primary bone marrow-derived dendritic cells [59].

The anti-inflammatory properties of tylophorine-based compounds have also been demonstrated in different murine models [54,59]. Particularly, the NK-007 analogue exerted a strong anti-inflammatory activity in dextran sulfate sodium-induced and acetic acid-induced colitis models [59], while the W-8 derivative (Figure 2B) significantly ameliorated ulcerative colitis induced by trinitrobenzene sulfonic acid in mice and increased Tregs in lymphoid tissues [57].

DCB-3503 was shown to inhibit the carrageenan-induced paw edema; similarly, synthetic dibenzoquinoline DBQ 33b (Figure 2B) significantly reduced carrageenan-induced rat paw edema along with the incidence and severity of collagen type II antibody-induced arthritis (CAIA), the gene expression of the arthritis inflammatory factors (such as *iNOS*, cyclooxygenase 2 or *COX2*, *TNFα*, and *IL-17A*), and the levels of *iNOS*, *c-Myc* (human homolog of the avian myelocytomatosis viral oncogene), and *HIF-1α* (hypoxia-inducible factor 1α). The ability of the compound to suppress protein expression of c-Myc and HIF-1α (associated with the Warburg effect) and their target genes by sequestering a ribonucleoprotein complex containing caprin-1 (cytoplasmic activation/proliferation-associated protein 1), the RNA-binding protein G3BP1 (Ras GTPase-activating protein-binding protein 1), and mRNAs of c-Myc and HIF-1α, has been hypothesized [54].

Among the mechanisms of anti-inflammatory activity, an interference of tylophorine (and its analogues) with the ribonucleoprotein (RNP) complex of caprin-1/G3BP1 and c-Myc mRNA seems to play a central role in the control of gene expression of several factors, which include pro-inflammatory and metabolic modulators; a similar mechanism has also been proposed for their antiproliferative activity [60].

**Figure 3:**
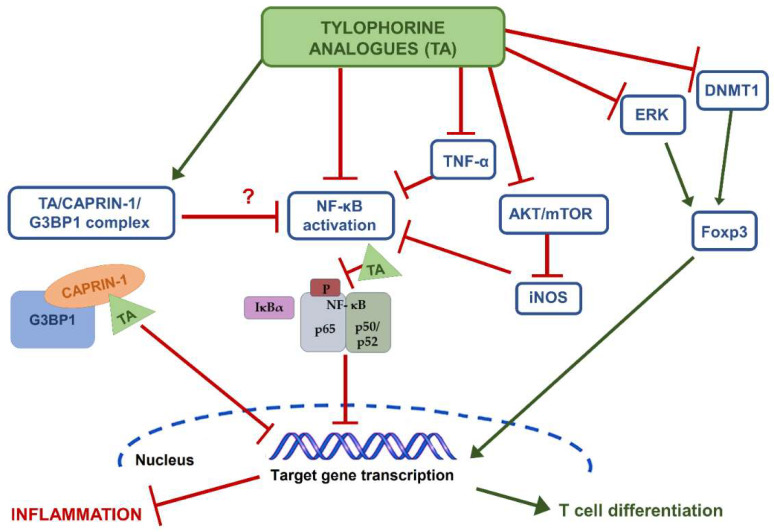
Schematic representation of the major mechanisms involved in the anti-inflammatory activity of tylophorine analogues. TA, tylophorine analogues; CAPRIN-1, cytoplasmic activation/proliferation-associated protein 1; G3BP1, Ras GTPase-activating protein-binding protein 1; NF-κB, nuclear factor kappa B; TNF-α, AKT, AK mouse plus Transforming or Thymoma; mTOR, mechanistic target of rapamycin kinase; iNOS, inducible nitric oxide synthase; ERK, extracellular signal-regulated kinase; DNMT1, DNA methyltransferase 1; Foxp3, Forkhead Box Protein P3.

Caprin-1 is a ubiquitous protein whose phosphorylated form regulates the normal cell cycle progression from G1 to S phase. It forms a complex with the nuclear transport factor 2-like function domain (NTF2) of G3BP1 within the cytoplasmic RNA granules and regulates the transport and translation of mRNA, which codifies proteins involved in cell proliferation and migration [60]. The carboxyl-terminal region of caprin-1 has been reported to selectively bind to c-Myc or cyclin D2 mRNAs. Qui et al. [60] suggested that tylophorine can directly bind to caprin-1, thus enhancing the recruitment of G3BP1, c-Myc mRNA, and cyclin D2 mRNA to form a tylophorine-targeted RNP complex further sequestered to the polysomal fractions, which led to the inhibition of protein expression (Figure 3).

Cells can also contain the RNA-binding protein G3BP2 (Ras GTPase-activating protein-binding protein 2), which is a paralog of G3BP1, carrying a similar NTF2-like domain, and other structural and functional differences; in neurons and glial cells, it specifically binds the IκBα/NF-κB complex, leading to its cytoplasmic retention and NF-κB inactivation [60]. NF-κB is a key factor in the inflammatory response to several stimuli, which include oxidative stress, diseases, and pathogens (e.g., viruses); therefore, its inactivation induces anti-inflammatory effects and benefits to inflamed tissues [61]. Although the exact inhibitory mechanisms of NF-κB by tylophorine analogues remain to be clarified, the available evidence suggests that these compounds can lead to its inactivation within a possible G3BP2/IκBα/NF-κB complex or by a TNF-α-inhibition.

### 2.4. Anticancer Activity

Tylophorine-based compounds, either naturally occurring or chemically synthesized, have been widely investigated for their potential anticancer power [26]. They exhibited marked antiproliferative and proapoptotic properties in vitro against numerous cancer cell lines (growth-inhibitory activity, GI50 of about 10 nM), with higher potency in melanoma and lung cancer [62]. For instance, tylophorine, tylocrebrine, and cryptopleurine inhibited protein synthesis in Ehrlich ascites tumor cells, with EC50 values ranging from 0.01 to 1 μM, while nucleic acid synthesis inhibition occurred at higher concentrations; protein and DNA synthesis inhibition by tylocrebrine was irreversible [29].

Furthermore, antofine and dehydroantofine exhibited potent antiproliferative activities in lung A549 and intestinal adenocarcinoma HCT-8 cancer cells, with EC50 values of about 1–2 ng/mL [50]. Similarly, DCB-3503 blocked the growth and differentiation of human pancreatic ductal PANC-1 and hepatocellular HepG2 cancer cells in xenograft mouse models [40]. The compound was also effective against drug-resistant cell lines, which overexpressed the MDR (multidrug resistance) and MRP (multidrug resistance-associate protein) transporters, and in Topo I down-regulated cells [62]. The trimethoxy derivatives of tylophorine, such as PF403 (Figure 2A), also exhibited promising antitumor properties in preclinical models [26].

Tylophorine has also been reported to inhibit angiogenesis via the VEGFR2 (vascular endothelial growth factor receptor 2) signaling pathway [63]. The anticancer activity was confirmed in tumor xenograft models, although with lower potency than in vitro studies, likely due to some pharmacokinetic issues [62]. Tylocrebrine showed promising anticancer effects in pre-clinical models; however, it failed phase I clinical trials due to the discovery of severe central nervous system side effects. In order to overcome this drawback, nanoparticle formulations have been developed, highlighting the possibility to retain the anticancer activity of tylocrebrine while reducing its brain penetration [64].

Several targets and enzymes involved in protein or nucleic acid synthesis (e.g., ribosome, thymidylate synthase, dihydrofolate reductase) have been associated with the antiproliferative activity of tylophorine compounds, although the fundamental target remains to be elucidated [29]. Gao et al. [62] suggested the involvement of a unique anticancer mechanism with respect to known anticancer molecules, which usually target DNA synthesis or induce DNA damage, resulting in cell cycle perturbations and the induction of apoptosis.

The inhibition of protein synthesis seems to be a central mechanism of tylophorine analogue anticancer activity, being more potently inhibited than nucleic acid synthesis [29]. Moreover, tylophorine analogues have been shown to block cell proliferation, inducing a G1 cell cycle arrest [62,65,66], which has been associated with a p53-stimulating transcription of the gene for the cyclin-dependent kinase inhibitory protein p21 [62] and with a c-Jun-mediated downregulation of cyclin A2 [66]. According to Yang et al. [66], tylophorine prolongs the NF-κB/PKCδ (protein kinase C delta)/MKK4 (mitogen-activated protein kinase kinase 4)/JNK (c-Jun N-terminal protein kinase) cascade involved in c-Jun phosphorylation and stabilization, as well as the PI3K (phosphatidylinositol 3-kinase)/PDK1 (pyruvate dehydrogenase kinase 1)/PP2A (protein phosphatase 2A)/eEF2 (eukaryotic translation elongation factor 2) signaling responsible for its translational blockade, thus leading to an increase in c-Jun expression, a downregulation of cyclin A2, and a promotion of G1 arrest. The inhibition of Akt (protein kinase B or PKB) and NF-κB pathways, along with the regulation of caspase 3 and 9 genes, have been found to be responsible for the apoptosis induced by tylophorine-based compounds [65].

DCB-3503 produced a marked NF-κB-inhibition and affected cancer cell survival by blocking the translation phase of protein synthesis through a down-regulation of pro-oncogenic and pro-survival proteins, such as cyclin D1 [45].

Qiu et al. [60] described a novel mechanism to explain the anticancer activity of tylophorine and its analogues: the compound can directly bind to caprin-1 to form a tylophorine-targeted ribonucleoprotein (RNP) complex with G3BP1, c-Myc mRNA, and cyclin D2 mRNA, which is sequestered to the polysomal fractions, with a subsequent block of the protein expression. Depletion of caprin-1 by tylophorine increased cancer cell resistance and decreased the formation of tylophorine-targeted RNP; moreover, c-Myc and cyclins D1/D2 are downregulated by the retinoblastoma tumor suppressor protein pRb (retinoblastoma protein), leading to cancer cell cycle block. This mechanism was shared by the tylophorine dibenzoquinoline-33b derivative and confirmed in xenograft models [60].

### 2.5. Toxicity Profile

Oral administration of *Tylophora indica* pure alkaloids at 1.25 and 2.5 mg/kg/day for 15 days was well tolerated in rats without toxicity signs; conversely, toxicity and mortality were registered at a dose of 5 mg/kg [67]. Cryptopleurine produced general toxic effects in vivo; conversely, tylocrebrine showed promising results in vivo, but produced central nervous system side effects (manifested as disorientation and ataxia) in clinical studies, likely because of its ability to cross the blood-brain barrier [29]. Tylophorine derivatives with decreased diffusion through the blood-brain barrier and improved water solubility displayed a more tolerable profile without neurotoxic effects [28,29,47].

## 3. Lycorine and Its Derivatives

### 3.1. Chemistry and Occurrence in Nature

**Figure 4:**
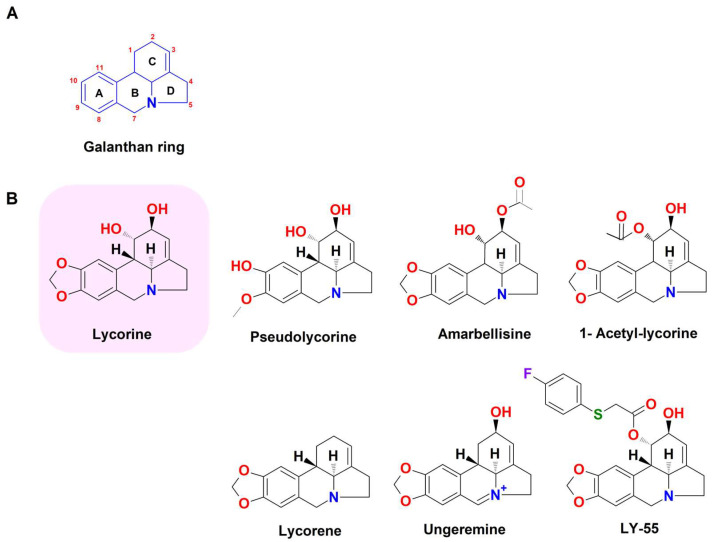
Chemical structure of lycorine-based alkaloids. (**A**) Galathan skeleton. (**B**) Lycorine derivatives (drawn by ChemSketch, ACD Labs Version 12.0).

Lycorine (C_16_H_17_NO_4_; molecular weight 287.31 g/mol; Figure 1) is a phenanthridine alkaloid containing a galanthan ring core (Figure 4A), and the bioactive component was firstly isolated from the bulbs of *Lycoris radiata* (L’Her.) Herb. (0.028% *w*/*w* amount), a plant belonging to the Amaryllidaceae family [68].

However, other species from the same botanical family contain lycorine, such as *Leucojum aestivum*, *Hymenocallis littoralis*, *Hippeastrum equestre*, *Clivia nobilis*, *Ammocharis coranica*, *Brunsvigia radulosa*, and *Crinum macowanii*, as shown in Table 3. Lycorine-analogs (Figure 4B), which usually possess an ortho-para coupling of a double bond in the C-ring [69], have also been identified in Amaryllidaceae species. Details about the compounds, their natural sources, and their chemical structures are displayed in Table 3 and Figure 4.

### 3.2. Antiviral Activity

Lycorine and various Amaryllidaceae isoquinoline alkaloids have been shown to inhibit a wide range of various viruses, such as flaviviruses (Japanese encephalitis, yellow fever, and dengue viruses), bunyaviruses, alphavirus, and lentivirus [95,96]. The lycorine ability to inhibit the replication of poliomyelitis virus [97], herpes simplex virus 1, Bunyamwera virus [98], West Nile virus, and SARS-CoV has also been reported by several studies [99].

Lycorine has also been shown to suppress the RNA replication and protein synthesis of the enterovirus 71 (EV71) virus, the avian influenza virus H5N1 [100], and the Zika virus through binding to the Zika RNA-dependent RNA polymerase (RdRp) protein [101]. It also markedly inhibited the influenza A virus H5N1 and delayed the export of nucleoprotein from the nucleus to the cytoplasm during replication [102]. Guo et al. [103] demonstrated that, among a total of 32 lycorine derivatives, 1-acetyl-lycorine (Figure 4B) was the most effective in suppressing EV71 and hepatitis C virus (HCV) replication in various cells. Further clarification of its activity through drug resistance analysis revealed that the compound targeted a phenylalanine (F76) in the EV71 2A protease binding site by stabilizing a zinc finger [102].

In the past years, the antiviral activity of lycorine against diverse coronaviruses, such as SARS-CoV, Middle East respiratory syndrome (MERS-CoV), HCoV-NL63, and HCoV-OC43, has been reported. Particularly, its ability to inhibit SARS-CoV-2 infection has been considered promising for the treatment of the COVID-19 pandemic [99,104].

Jin et al. [105] studied the antiviral effect of lycorine as a non-nucleoside antiviral agent (NNA) on emerging coronaviruses, such as SARS-CoV, MERS-CoV, and SARS-CoV-2, and highlighted remarkable inhibitory effects on RdRp of the CoVs in a cell-based reporter assay. The compound was also found to be nontoxic up to 10 μM in HEK293 cells [104]. Accordingly, it inhibited the MERS-CoV infection in Vero cells with 2.1 μM IC50 (50% inhibitory concentration) and higher than 50 μM CC50 (50% cytotoxic concentration). Data indicated that lycorine directly inhibits MERS-CoV RdRp activity with an IC50 of 1.4 μM without affecting the host’s transcriptional and translational machinery; it was also more potent than remdesivir (6.3 μM IC50), a known inhibitor of MERS-CoV RdRp activity [106].

The antiviral activity of lycorine against SARS-CoV and SARS-CoV-2 infections in Vero cells was also examined [104]. Data showed that the IC50 values of lycorine antiviral activity were 1.0 μM and 0.9 μM against SARS-CoV and SARS-CoV-2, respectively. Similarly, the IC50s of remdesivir were 4.1 and 6.5 μM against SARS-CoV and SARS-CoV-2 viruses, respectively, thus confirming the higher potency of lycorine.

Ren et al. [107] constructed a three-dimensional model of the human ribosome in complex with lycorine. Analysis of the stable complex showed that lycorine fits snugly into the peptidyl transfer site and was further stabilized by forming hydrogen bonds with A4397, U4450, U4446, and G4393.

A key translational recoding mechanism of SARS-CoV-2 consists of a -1 programmed ribosomal frameshifting (−1 PRF), stimulated by a structured RNA motif of the SARS-CoV-2 genome. Frameshift stimulation element (FSE) as a structured RNA motif includes a slippery site′ UUUAAAC in the 5′heptanucleotide and a three-stem pseudoknot [108]. Previous studies showed that the RNA pseudoknot could be considered a potential target for developing small-molecule antiviral agents via attenuating viral propagation.

Lycorine has been found to bind strongly to the position near the slippery site and FSE [107]. A salt-bridge interaction formed between the amino group of lycorine and the phosphodiester linkage of U13 assists in the formation of a complex between the compound and RNA. This binding is further stabilized by a hydrogen bonding network between the two hydroxyl groups of lycorine and ribonucleotides. Conversely, lycorine did not bind to RdRp in a concentration-dependent manner in the absence of RNA but presented an apparent strong interaction with RdRp in the presence of RNA.

**Figure 5:**
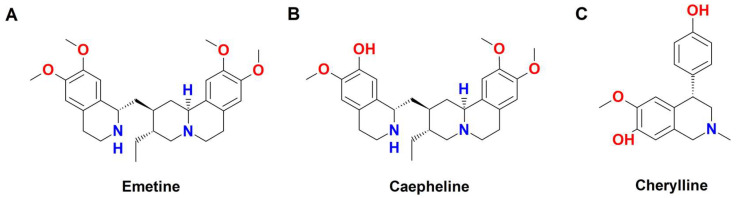
Chemical structure of the isoquinoline-based alkaloids emetine (**A**), cephaeline, (**B**) and cherylline (**C**) (drawn by ChemSketch, ACD labs Version 12.0).

By comparison, the isoquinoline-based alkaloids emetine and cephaeline (Figure 5A,B), which also showed interesting antiviral properties [16], were evaluated in the same experimental model. 

They were found to bind with nsp-12 with a dissociation constant (KD) of 25.7 μM and 19.6 μM, respectively, and a ten-fold increase in binding affinities of these two compounds was observed in the system of RdRp with the presence of RNA. At first sight, it seems that lycorine is too small to occupy the extremely large cavity of RdRp. However, the binding of RNA to RdRp makes the cavity of RdRp compact and favors the binding of these alkaloids, especially the smaller alkaloid lycorine. Correspondingly, the binding affinities come up to 8.3 μM for lycorine, 8.0 μM for emetine, and 8.9 μM for cephaeline [107]. These results indicated that the inhibition mechanism of emetine and cephaeline was a bit different from that of lycorine in SARS-CoV-2 infection. On the other hand, emetine and cephaeline potently inhibited SARS-CoV-2 replication and transcription by blocking the formation of the RNA template-product duplex or occupying the RNA growth site, whereas lycorine inhibited RNA synthesis by occupying the RNA growth site [107].

The binding cavity of nucleocapsid protein (N protein) is relatively large and flat, and therefore, it is very challenging to design strongly bound molecules; therefore, relatively weak binding affinities can be expected by binding the three alkaloids emetine, cephaeline, and lycorine to the N protein. In fact, the obtained binding affinities of emetine, cephaeline, and lycorine binding with nucleocapsid proteins were determined to be 18.6, 53.8, and 58.2 μM, respectively.

Computational studies revealed that all three alkaloids are able to form a π-π interaction with Y109 of the N protein, and additional hydrogen bonds were formed between emetine as well as cephaeline and the residues of T91 and Y109 of the N protein. An extra hydrogen bond was also found for lycorine with R149. Therefore, blocking the recognition between the viral genome and N protein was suggested as a possible mechanism for the three alkaloids, which may interfere with viral core assembly and maturation [107].

The antiviral efficacy of lycorine, emetine, and cephaeline against SARS-CoV-2 in Vero E6 cells was further evaluated by quantifying the viral copy numbers in the cell supernatant via qRT-PCR [107]. The results showed that the three alkaloids dose-dependently inhibited SARS-CoV-2 replication with 0.4, 0.008, and 0.01 μM IC50 values, respectively. As revealed by the CCK8 (Cell Counting Kit 8) assay, lycorine, emetine, and cephaeline showed diverse cytotoxicity profiles in Vero E6, with CC50 values > 1000, 2.2, and 49.0 μM, respectively. The cytotoxicity of lycorine, emetine, and cephaeline was also determined in HEK293T cells and Huh-7 cells, and the CC50 values were 1.0, 0.04, and 1.9 μM and 0.8, 0.03, and 3.0 μM, respectively [107]. Altogether, these findings showed that lycorine, emetine, and cephaeline possess interesting antiviral activities, albeit with some concerns due to their toxicity, which is usually higher for emetine and cephaeline [106]. The toxic effects of these alkaloids are largely caused by their interactions with the human ribosomes; therefore, structural modifications to selectively decrease the binding affinities of these compounds with ribosomes could be further approached to harness their antiviral power while limiting cytotoxicity [107]. 

According to their binding sites on the ribosome, they concluded that lycorine inhibits protein synthesis by interfering with peptide bond formation, while blocking the E-site of the ribosome by emetine stops protein synthesis. The computational studies also indicated that these positively charged alkaloids could strongly bind to the negatively charged −1 programmed ribosomal frameshifting (−1 RPF) of SARS-CoV-2, which contributes to blocking the propagation of the virus [107].

The multiple-target activity mechanisms of the three studied alkaloids were confirmed by the smaller effective concentration in the cell-based viral infection assay than the molecular-level assay. Previous animal toxicological experiments exhibited low toxicity and mild side effects of lycorine [109]. According to the previous literature, lycorine is able to penetrate the CNS (central nervous system) by crossing the blood-brain barrier, which suggests that it has the potential to treat SARS-CoV-2 infections in the brain [110].

In order to find potent and broad-spectrum inhibitors of coronaviruses, Shen et al. [111] performed a high-throughput screening of a 2000-compound library of approved drugs and pharmacologically active compounds using the established genetically engineered human CoV-OC43 (HCoV-OC43) strain, expressing Renilla luciferase (rOC43ns2Del-Rluc), to infect BHK-21 cells. They identified seven compounds, which include lycorine and emetine, as broad-spectrum inhibitors. A primary screening revealed that 56 hits significantly inhibited rOC43-ns2Del-Rluc replication activity by more than 70% at a concentration of 10 µM, with less than 80% cytotoxicity. To exclude the possibility that the observed antiviral activity was specific to rOC43-ns2Del-Rluc, the antiviral activity of the 56 hits against wild-type HCoV-OC43 (HCoV-OC43-WT) was assessed by quantitative reverse transcription (qRT)-PCR at a 5 µM concentration, which resulted in the selection of 36 compounds. Subsequently, 17 compounds inhibited the replication of HCoV-NL63 (alpha-CoV that usually causes the common cold) with EC50 values lower than 5 µM, whereas 13 and 12 compounds inhibited MERS-CoV and MHV-A59 replication with EC50 values lower than 5 µM. Altogether, these results showed that both lycorine and emetine inhibited the replication of all CoVs with EC50 values lower than 5 µM and significantly suppressed HCoV-OC43 replication [111].

Lycorine showed a potent anticoronaviral activity, with EC50 values ranging from 0.15 µM to 1.63 µM; moreover, its selective index (SI) for HCoV-OC43 was 29.13, indicating a potent activity [111].

It is proven that HCoV-OC43 crosses the blood-brain barrier, infects the CNS, and causes neurological disorders; the virus also replicates rapidly in the brain and causes encephalitis and death in mice [110].

In order to evaluate the effect of the compounds against the HCoV-OC43-induced CNS infection, female BALB/c mice were infected via the intranasal route with HCoV-OC43-WT and treated with the seven inhibitors for 14 days, and their survival was monitored for up to 20 days. In this experiment, 30 mg/kg of chloroquine was used as a positive control [110]. All mice in the phosphate-buffered saline (PBS) DMSO-treated group died within 6 days after the HCoV-OC43-WT infection. Successfully, 83.3% of mice in the lycorine-treated group could live for 20 days after inoculation, equivalent to the survival rate of the chloroquine-treated group. Correspondingly, the viral loads in the brain and spinal cord were not detectable in the lycorine-treated group, and HCoV-OC43 nucleocapsid protein was only found by immunohistochemistry in the PBS-DMSO-treated group and not in the lycorine-treated group [111]. The real-time monitoring of the effect of lycorine on the spread and replication of HCoV-OC43 in the mouse central nervous system showed that PBS-DMSO-treated mice gradually increased the signal intensity in bioluminescence imaging, whereas no signal was detected in the brains of lycorine-treated mice post-inoculation.

Min et al. [112] generated a cell-based SARS-CoV-2 RdRp activity assay by modifying a previously established model to screen for SARS-CoV-2 RdRp inhibitors. They examined whether lycorine could inhibit SARS-CoV-2 RdRp activity by treating the cells with the compound for 15 h, then transfecting with p(+)FLuc-(-)UTR-NLuc and pCI-SARS-CoV-2 nsp12-N-term Flag (pCI-SARS2-nsp12N) and measuring the SARS-CoV-2 RdRp activity. Lycorine dose-dependently reduced the NLuc (nanoLuc luciferase) activity, whereas the FLuc (firefly luciferase) value remained constant.

Moreover, it completely inhibited the SARS-CoV-2 RdRp activity at 4.4 µM [110], with a 1.5 µM IC50 value, thus suggesting a higher potency with respect to the standard RdRp inhibitor remdesivir.

Wang et al. [113] evaluated the antiviral properties of the lycorine derivative LY-55 (2-hydroxy-2,3a,4,5,7,12b-hexahydro-1H[1,3]dioxolo[4,5-j]pyrrolo[3,2,1-de]phenanthridin-1-yl-2-((4-fluorophenyl)thio)acetate hydrochloride; C_24_H_22_FNO_5_S.HCl, molecular weight 491.96g/mol; Figure 4B) against Enterovirus 71 (EV71) and Coxsackievirus A16 (CVA16). LY-55 showed a higher therapeutic index than that of lycorine, with 106.4 and 77.3 µM TC50 (50% toxic concentration) values in Vero cells, respectively. It also provided partial protection to mice from EV71 infection, as indicated by the decreased viral load and protein expression levels in muscles, clinical scores, and increased survival rates of infected mice; however, it was not directly virucidal [112].

Enterovirus 71 infection has been reported to induce autophagy to promote EV71 replication in vivo and in vitro. The JNK signaling pathway is closely related to autophagy and plays an important role in the regulation of cell growth, proliferation, differentiation, migration, and apoptosis; therefore, its inhibition could inhibit autophagy [112]. Therefore, inhibition of autophagy may be a promising strategy to inhibit the replication of EV71 and CVA16. In this respect, Wang et al. [112] showed that LY-55 could effectively reduce JNK phosphorylation and inhibit the autophagy induced by EV71 and CVA16 infection; indeed, the LC3II (microtubule-associated protein light chain 3 II) autophagy marker was found to decrease after treatment with LY-55 and lycorine. On the other hand, inhibiting autophagy blocked the degradation of ubiquitin-binding protein p62 and increased its protein level, thus corroborating the autophagy inhibition by LY-55. At last, the expression of the EV71 VP1 protein was significantly decreased by LY-55 treatment while increasing the survival rates of EV71-infected mice.

Ka et al. [113] studied the ability of a crude extract from the bulbs of *Crinum jagus*, containing lycorine along with other compounds such as the isoquinoline-based alkaloid cherylline (Figure 5C), to inhibit Dengue virus (DENV), a flavivirus belonging to the family Flaviviridae and related to Zika virus (ZIKV). The extract (from 0.078 to 2.5 ug/mL) showed to inhibit the DENV infection with a weak cytotoxicity in Huh7 cells, achieving about a 29% lowering of cell viability at the highest tested concentration. The treatment with lycorine effectively decreased the DENV-GFP infection, thus confirming its strong anti-DENV activity; moreover, a remarkable antiviral activity was highlighted for the purified cherylline. The antiviral activity of lycorine and cherylline occurred at nontoxic concentrations, thus strengthening interest in these compounds as possible novel therapeutic strategies.

Structure-activity analysis of the antiviral effects of lycorine has shown that the free hydroxyl groups at C1 and C2, the intact benzodioxole group at the A-ring (Figure 4A), the basic nitrogen, and the C3-C4 double bond are crucial for its optimum activity [114].

### 3.3. Anti-inflammatory Activity

High-mobility group box1 (HMGB1), a key regulator of acute lung injury (ALI), is known to mediate the activation of the TLR4 (toll-like receptor 4)/NF-κB pathway; moreover, the block of this pathway has been shown to relieve LPS-induced acute lung injury. Previous evidence has reported that lycorine is able to inhibit HMGB1 expression in cancer progression [69].

Ge et al. [115] showed that after LPS treatment, the lung injury score, lung wet-to-dry weight ratio, and malondialdehyde (MDA) production in the lung tissues and the expression levels of tumor necrosis factor-α, interleukin-1β, and interleukin-6 in bronchoalveolar fluid were significantly increased, whereas lycorine decreased their levels. They also found that the HMGB1/Toll-like receptors (TLRs)/NF-κB pathway was activated by LPS injection, while lycorine attenuated the activity of the HMGB1/TLRs/NF-κB pathway in the lung tissues of the treated mice [114]. Interestingly, in groups treated with LPS and lycorine, the protein levels of HMGB1, TLR4, TLR5 (toll-like receptor 5), Myd88 (myeloid differentiation primary response protein 88), PP65 (phosphoprotein 65), and P-IκBα (phosphorylated IκBα) were markedly decreased and the IκBα level was increased, compared with the LPS group.

In vitro studies also showed that the levels of inflammatory cytokines and MDA content were significantly decreased by lycorine, which also attenuated the activity of the HMGB1/TLRs/NF-κB pathway in LPS-treated MLE-12 cells. Moreover, their results showed that lycorine is very similar to glycyrrhizic acid in alleviating the inflammatory response and oxidative stress in LPS-induced lung injuries, and the simultaneous use of lycorine and glycyrrhizic acid has a synergistic effect in the treatment [114]. Blocking the HMGB1/TLRs/NF-κB pathway by lycorine to alleviate LPS-induced lung injury from inflammation and oxidative stress gave a new perspective for acute lung injury (ALI) therapy to be harnessed clinically.

Chen et al. [115] showed that lycorine (LY) suppressed in vitro the interleukin-1β (IL-1β)-induced synthesis of the MMP-3 and MMP-13 matrix metalloproteinases. MMPs are key degrading enzymes of the extracellular matrix, critically involved in the progression of inflammatory processes such as osteoarthritis (OA). In fact, inflammatory mediators result in the production of IL-1β and TNF-α in chondrocytes and the secretion of mononuclear inflammatory cells. These inflammatory cytokines stimulate the expression of proteases, including MMPs. MMPs are the major mediators of cartilage turnover in OA and are able to degrade all components of the cartilage in the extracellular matrix. Collagen turnover is almost exclusively mediated by MMPs with collagenolytic abilities, including MMP-1 and MMP-13. The synthesis of MMPs is predominantly regulated at the transcriptional level by activator protein 1 (AP-1) and nuclear factor (NF)-κB. The binding activity of AP-1 is regulated by IL-1β and TNF-α through signaling pathways. Jun N-terminal kinase (JNK) can phosphorylate c-Jun to activate the AP-1 DNA-binding complex and promote the expression of MMPs.

**Figure 6:**
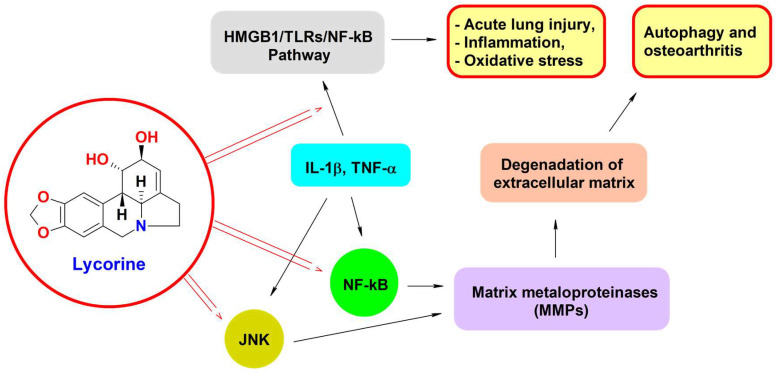
A schematic representation of the biochemical signaling pathways involved in the induction of pathologic conditions such as acute lung injury, inflammation, autophagy, and osteoarthritis. Lycorine inhibits the pathway, as indicated by the arrows. HMGB1, high-mobility group box1; TLRs, toll-like receptors; NF-κB, nuclear factor kappa B; JNK, c-Jun N-terminal protein kinase; IL1β, interleukin 1β; TNF-α, tumor necrosis factor α.

Molecular analysis, carried out by Chen et al. [115], revealed that lycorine also inhibited the phosphorylation of c-JNK and the activation of NF-κB by IL-1β. Therefore, the anti-inflammatory activity of lycorine seems to arise from its ability to affect JNK and NF-κB pathways, thus blocking the IL-1β-induced expression of MMP-3 and MMP-13, as shown in Figure 6. Based on these findings, a possible usefulness of this compound in the treatment of OA has been hypothesized.

### 3.4. Anticancer Activity

**Figure 7:**
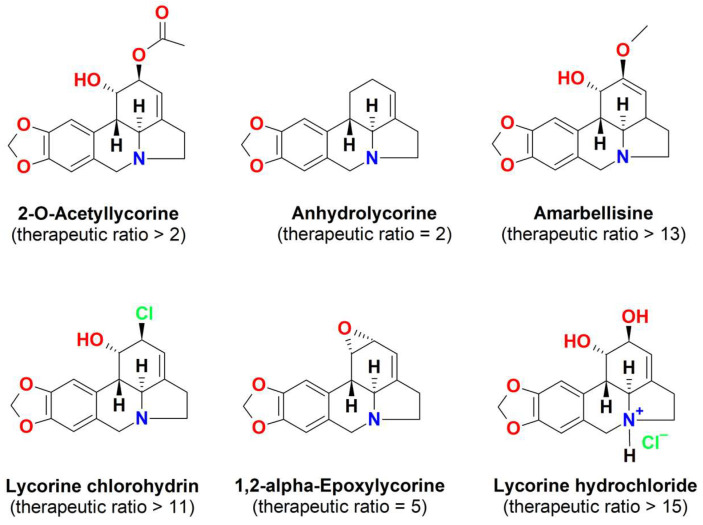
Chemical structures of lycorine derivatives evaluated for their in vitro anticancer activity.

Lamoral-Theys et al. [116] evaluated the anticancer and safety potential of lycorine and its analogues (Figure 7). 

Lycorine exhibited marked growth inhibitory activity in all the cancer cell lines, including A549, U373, OE21, SKMEL-28, Hs683, and B16F10, and its anticancer activity was higher than the analogues [116]. Moreover, it showed the highest in vitro therapeutic ratio over a panel of six cancer cell types and three normal cell types, being at least 15 times more cytotoxic in cancer cells than the normal cells. Amarbellisine (Figure 7), one of the lycorine analogues, displayed a similar safety profile to lycorine, while anhydrolycorine (Figure 7), which exhibited an antitumor power like that of lycorine, displayed a weaker therapeutic ratio than lycorine [114].

Over the years, the anticancer power of lycorine and its derivatives has been reported in diverse in vitro and in vivo cancer models [117,118,119,120,121,122,123,124,125,126,127,128]. It has also been found to affect cell proliferation in drug-resistant cancer cells [69,124,129]. A modulation of signal transducer and activator of transcription 3 (STAT3) protein, RNA binding motif 10 (RBM10), NF-κB, and the E3 ubiquitin ligase NEDD4-1 (neural precursor cell expressed developmentally down-regulated protein 4-1), along with the induction of apoptosis and cell cycle arrest, have been associated with its anticancer properties [123,130,131]. Its power has been considered promising in cancer research, although further studies are needed to better understand its mechanisms of action and clinical efficacy.

Structure-activity relationship studies highlighted that the hydroxyl groups at C1 and C2 and the benzodioxole group of the galanthan skeleton (Figure 4A) are also related to its cytotoxicity. The disubstitution, especially the diacetylation of the free hydroxyl groups at C1 and C2 and/or the degradation of the benzodioxole group, should markedly reduce the cytotoxicity of the lycorine derivatives [114].

## 4. Discussion and Future Perspectives

Pharmacological research on viral infections remains an ongoing challenge for scientists. Viruses are constantly evolving and adapting, giving rise to new strains and variants that can evade existing treatments. As a result, drug discovery and the development of novel antiviral therapies remain critical endeavors in safeguarding public health and mitigating the impact of viral outbreaks. The recent pandemic of SARS-CoV has strengthened the need for novel, effective, low-toxic, and affordable bioactive agents to counteract the viral infection and its complications [132,133,134,135,136]. In this context, marine and plant-derived alkaloids were investigated as possible candidates for COVID-19 treatment owing to their wide variety of structures and remarkable bioactivities [24,27].

In the present study, we outlined the potential interests of tylophorine and lycorine, two plant-derived alkaloids that share a common benzoindolizidine scaffold. These compounds are mainly identified in tropical and subtropical plant species, belonging to the Ascepidaceae and Amaryllidaceae species [29,69].

These alkaloids and their analogues and derivatives were found to be endowed with powerful and broad-spectrum antiviral properties; moreover, they were able to counteract inflammation, which often underpins the complications of viral diseases [29,69]. At last, their anticancer properties hold promise not only for advancing cancer research but also for mitigating the oncogenic effects of viruses [29,69]. This evidence suggests that the substances could effectively counteract the pathogenesis of respiratory viral disease and its harmful effects. Regarding the structural activity relationship, the nonplanarity of the benzoindolizidine scaffold seems to be a basic feature to ensure the bioactivity of tylophorine derivatives; moreover, the presence of hydroxyl groups at C14 and C3 potentiates their antiviral effects [48]. Conversely, the presence of the dioxole ring, the hydroxyl groups, and a basic nitrogen are structural parameters that contribute to the activity of lycorine-based alkaloids [69].

Providing both antiviral and anticancer abilities through tylophorine and lycorine alkaloids is noteworthy and promising for addressing various clinical scenarios. Indeed, cancer patients are highly susceptible to viral diseases, and chemotherapy often leads to an increased risk of their worsening because of an impairment of their immune defenses [137,138]. In this context, the antiviral properties of tylophorine and lycorine alkaloids may counteract the development of viral infections and lower their severity in immunocompromised cancer patients, also supporting the chemotherapy efficacy; moreover, they may reduce the risk of cancer relapses in patients with chronic viral infections, acting as potential antiviral chemopreventive agents. In addition to their antiviral and anticancer activities, these alkaloids possess anti-inflammatory properties. This confers the added benefit of preventing complications arising from the cytokine storm triggered by viral infections, such as SARS-CoV-2, and inhibiting the development of an inflammatory environment, which is often a contributing factor in cancer progression and invasion [139].

Despite these promises, several challenges, such as the safety and bioavailability of these substances, need to be addressed. For instance, the phenanthroquinolizidine-based tylophorine derivatives possess a marked antiviral potency [46], but are highly toxic in vivo [28]. Moreover, neurotoxicity is the major limit to the pharmaceutical development of phenanthroindolizidine-based tylophorine analogues, especially tylocrebrine. Interestingly, highly water-soluble synthetic derivatives of tylocrebrine, with decreased diffusive abilities through the blood-brain barrier, retained and improved the antiviral and anti-inflammatory effects of the lead compound, also limiting its toxicity risk [28,29,47]. This suggests that future medicinal chemistry interventions may be decisive to develop safer and highly effective antiviral derivatives of tylophorine.

Lycorine possesses a low toxic profile [109], along with a wide and powerful antiviral power and anti-inflammatory properties [69], thus suggesting promising applications to counteract the pathogenesis and complications of SARS-CoV-2. Indeed, it was found capable of inhibiting RdRp and binding to the ribosomal RNA to block protein synthesis; moreover, it reduces JNK phosphorylation and inhibits matrix metalloproteinase enzymes and autophagy [69]. At last, blocking the HMGB1/TLRs/NF-κB pathway may potentially reduce the release of inflammatory cytokines, thus limiting the inflammation responsible for the complications of viral infections.

Lycorine has also been reported to penetrate the CNS [110], albeit being well tolerable; this suggests a possible application to treat COVID-19-related CNS complications [69]. However, further studies are needed to confirm the lack of harmful central effects of lycorine and its clinical efficacy.

An essential consideration regarding both tylophorine and lycorine pertains to their pharmacokinetics. Despite the extensive array of pharmacological investigations, these aspects have been less investigated. Preclinical studies revealed that lycorine was extensively distributed in all tissues, becoming undetectable within 2 h post-injection. Furthermore, it demonstrates rapid accumulation in the kidneys and liver, suggesting potential elimination through excretory organs and liver metabolism [140,141,142]. More comprehensive studies should be encouraged to elucidate the bioavailability, metabolism, and tissue distribution of these compounds and to better understand their safety and toxicological features, thus allowing their possible therapeutic applications.

Furthermore, various issues concerning the pharmaceutical development of natural substances warrant attention. These include sustainable sourcing from natural reservoirs, enhancing bioavailability and stability through appropriate delivery systems, considerations of patentability, and the imperative for rigorous pharmacological investigations. The availability of natural sources containing these substances is of paramount importance, both for direct utilization and for subsequent medicinal chemistry endeavors. Ensuring a responsible use of known plant species and limiting their overexploitation is crucial; moreover, phytochemical and pharmacognostic research to explore potential innovative high-yield and eco-friendly resources, such as waste biomass, should be encouraged. Biotechnological productions and innovative cultivation and extraction technologies may also be considered [143].

Altogether, the collected evidence highlights a possible interest in tylophorine and lycorine as antiviral and/or adjuvant strategies and encourages future, more in-depth preclinical and clinical investigations to overcome their drawbacks and harness their power for therapeutic purposes.

## Figures and Tables

**Table 1:** Natural sources of the major tylophorine-based alkaloids.

	Species/Plant Part	Family	Ref.
Tylophorine	*Tylophora indica* (Burm. F.) Merr./leaves and root	Asclepidaceae	[31]
	*Tylophora crebriflora* S.T. Blake/whole plant	Asclepidaceae	[37]
	*Tylophora tanakae* Maxim. Ex Franch. & Sav./leaves	Asclepidaceae	[38]
	*Tylophora mollissima* Wt./whole plant	Asclepidaceae	[39]
Tylophorinine	*Tylophora indica* (Burm. F.) Merr./leaves and root	Asclepidaceae	[31]
	*Tylophora atrofolliculata* Wt./whole plant	Asclepidaceae	[40]
	*Tylophora mollissima* Wt./whole plant	Asclepidaceae	[39]
Tylophorinidine	*Tylophora indica* (Burm. F.) Merr./leaves and root	Asclepidaceae	[31]
Tylocrebrine	*Tylophora crebriflora* S.T. Blake/whole plant	Asclepidaceae	[37]
Antofine	*Ficus septica* Burm. F./stems	Moraceae	[41]
	*Cynanchum komarovii* Al. Iljinski/aerial parts	Asclepidaceae	[42]
Cryptopleurine	*Boehmeria caudata* (L.) Sw./stem wood with bark	Urticaceae	[43]
	*Cryptocarya laevigata* Blume/stem bark	Lauraceae	[43]

**Table 3:** Natural sources of lycorine and its major analogues.

	Species/Plant Part	Family	Refs.
Lycorine	*Amaryllis belladonna* L.	Amaryllidaceae	[70]
	*Ammocharis coranica* (Ker Gawl.) Herb.	Amaryllidaceae	[71]
	*Brunsvigia radulosa*	Amaryllidaceae	[72]
	*Clivia nobilis* Lindl.	Amaryllidaceae	[73]
	*Crinum asiaticum var. japonicum* Bak.	Amaryllidaceae	[74]
	*Crinum macowanii* Bak.	Amaryllidaceae	[75,76]
	*Galanthus nivalis* L.	Amaryllidaceae	[77]
	*Hippeastrum equestre* Herb.	Amaryllidaceae	[78]
	*Hippeastrum solandriflorum* Herb.	Amaryllidaceae	[79]
	*Hymenocallis littoralis* (Jacq.) Salisb.	Amaryllidaceae	[80]
	*Leucojum aestivum* L.	Amaryllidaceae	[81,82]
	*Lycoris radiata* (L’Her.) Herb./bulbs	Amaryllidaceae	[68,83]
	*Narcissus tazetta* L.	Amaryllidaceae	[84,85]
Pseudolycorine	*Hymenocallis littoralis* (Jacq.) Salisb.	Amaryllidaceae	[86]
	*Hymenocallis littoralis* (Jacq.) Salisb.	Amaryllidaceae	[86]
	*Leucojum aestivum* L.	Amaryllidaceae	[87]
	*Lycoris guangxiensis* Y-Xu & G.J. Fan	Amaryllidaceae	[88]
	*Lycoris radiata* (L’Her.) Herb./bulbs	Amaryllidaceae	[89]
	*Zephyranthes grandiflora* Lindl.	Amaryllidaceae	[90]
Amarbellisine	*Amaryllis belladonna* L.	Amaryllidaceae	[70]
	*Zephyranthes candida* (Lindl.) Herb.	Amaryllidaceae	[91]
Ungeremine	*Crinum asiaticum var. japonicum* Bak.	Amaryllidaceae	[74]
	*Crinum x amabile* Donn. Ex Ker Gawl.	Amaryllidaceae	[92]
	*Galanthus nivalis* L.	Amaryllidaceae	[92]
	*Hymenocallis littoralis* (Jacq.) Salisb.	Amaryllidaceae	[92]
	*Hippeastrum solandriflorum* Herb.	Amaryllidaceae	[92]
	*Narcissus tazetta* L.	Amaryllidaceae	[85]
	*Nerine bowdenii* W. Watson	Amaryllidaceae	[93]
	*Zephyranthes grandiflora* Lindl.	Amaryllidaceae	[90]
Lycorene	*Caliphuria tenera* Bak.	Amaryllidaceae	[94]

## Data Availability

Not applicable.
